# Cognitive Linguistics: Analysis of Mapping Knowledge Domains

**DOI:** 10.3390/jintelligence10040093

**Published:** 2022-10-24

**Authors:** Ahmed Alduais, Ammar Al-Khawlani, Shrouq Almaghlouth, Hind Alfadda

**Affiliations:** 1 Department of Human Sciences, University of Verona, 37129 Verona, Italy; 2 Institute of English Studies, University of Warsaw, 00-927 Warsaw, Poland; 3 Department of English, King Faisal University, Al-Ahsa 31982, Saudi Arabia; 4 Department of Curriculum and Instruction, King Saud University, Riyadh 11362, Saudi Arabia

**Keywords:** cognitive linguistics, cognitive processes, perceptual processes, perception, mental representation, conceptual representation, scientometric review

## Abstract

Language acquisition, processing, comprehension, and production encompass a complex mechanism. Particularly, the mechanisms by which we make sense of language, including perception, conceptualization, and processing, have been controversial topics among cognitive linguists and researchers in cognitive sciences. Cognitive processes such as attention, thought, perception, and memory play a significant role in meaningful human communication. This study aimed to apply the science mapping method to detect and visualize emerging trends and patterns in literature pertaining to cognitive linguistics. In order to accomplish this, eight bibliometric and eight scientometric indicators were used in conjunction with CiteSpace 5.8.R3 and VOSviewer 1.6.18 for scientometric analysis and data visualisation. The data were collected and triangulated from three databases, including 2380 from Scopus, 1732 from WOS, and 9911 from Lens from 1969 to 2022. Among the findings were the visualization of eight bibliometric indicators regarding the knowledge production size of cognitive linguistics based on year, country, university, journal, publisher, research area, authors, and cited documents. Second, we presented scientometric indicators with regard to cognitive linguistics development, including the most important authors in the field, co-citation networks, citation networks, sigma metrics to detect works with potential citation growth, and clusters to group related topics to cognitive linguistics. We conclude the study by emphasizing that cognitive linguistics has evolved from the micro level where it focused on studying cognitive aspects of language in relation to time, language, and modality dimensions, to the macro level, which examines cognitive processes and their relationship to the construction of meaningful communication using both sensation and perception.

## 1. Introduction

### 1.1. The Rise of Cognitive Linguistics

Language is inherently dynamic and structured (Hartmann 2021; Langacker 2016, p. 143); and linguistic theories throughout history have been occupied with decoding it as such. While these linguistic theories varied deeply, categorizing their differences can be approached from different perspectives. Perhaps one of the simplest of these is by examining their epistemological and theoretical take of grammatical structure as well as how much they envision meaning within such conception (Winters and Nathan 2020). With this in mind, two opposing theories appear instantly, Generative Grammar and its exclusive fixation of formal structure and Cognitive Linguistics which was envisioned initially within works on Generative Grammar but developed further to become a rather rival opponent. The latter, Cognitive Linguistics, is the focal point of this detailed review as an ever-expanding enterprise.

Cognitive Linguistics is a flexible framework with no single or uniform doctrine (Geeraerts and Cuyckens 2007); no ‘central gurus’ or ‘crystalized formalism’ (Janda 2006, p. 5). Instead, it is a multidisciplinary enterprise (Grygiel and Kieltyka 2019) seeking to highlight the assumption that linguistic abilities are deeply rooted within human cognition. Within such conception, meaning is central and grammar is usage-shaped (Dąbrowska and Divjak 2015). This strong affiliation between language and cognition, defining the essence of Cognitive Linguistics, perceives cognition as the way in which humans are able to organize and interact with different objects and events in the world, including how we shape ourselves across different dimensions (Grygiel and Kieltyka 2019). This results in the instrumental construction of language as a tool utilizing the categorization and processing of overlapping linguistic units within human experience (Geeraerts and Cuyckens 2007). 

As an acknowledged field of study, Cognitive Linguistics emerged in the middle of the second half of the twentieth century; however, it has roots that can be traced back much further. Nerlich and Clarke (2001) contend to the long past that Cognitive Linguistics has as opposed to its relatively short history. Since its infancy, it has been always possible to see the strong links between this new linguistic field and works of Gestalt psychology (Evans and Green 2006)—according to which, the whole is more than the sum of its parts (Winters and Nathan 2020)—as well as other cognitive sciences. Cognitive Linguistics also reveals historical connections to pre-structuralist nineteenth century literature as exhibited in the work of philologists, Michel Bréal (1832–1915), on meaning as a psychological and mental process (Grygiel and Kieltyka 2019) as well as to modern cognitive psychology through the work of psychologist Eleanor Rosch’s prototype theory in the mid-1970s (Winters and Nathan 2020). It also exhibits some similarities with functional linguistics which flourished around the same time (Nuyts 2007). 

It was the dominance of the behaviorist and strictly structuralist perceptions along with their emphasis on extreme empiricism in the 1950s and 1960s that led to the birth of Cognitive Linguistics (Divjak et al. 2016). To illustrate, generative grammar, in particular, along with its focus on nativism, led to some dissatisfaction within linguists (Dąbrowska and Divjak 2015) that consequently fueled an opposing stance of language in which the complete separation between language and cognition proposed within the Chomskyan approach was rejected. On the contrary, language is perceived as an integral component of human cognition (Grygiel and Kieltyka 2019; Geeraerts and Cuyckens 2007) in which usage is centralized with an increasing interest in introspection as a primary source of evidence (Divjak et al. 2016). This was evident in what was later coined as the linguistics wars by Harris (1993), in which Lakoff, who was initially supportive of generative grammar, transformed Chomsky’s standard theory into generative semantics (Tay 2014). However, later on, Lakoff decided to abandon generative semantics and adopt the term Cognitive Linguistics instead (Nerlich and Clarke 2007); the earliest reference of which was in a paper by Lakoff and Thompson (Lakoff and Thompson 1975). By the same token, Langacker (Langacker 1978) was working on his theory of space grammar, but also decided to abandon this term in favor of Cognitive Linguistics. This was followed by Lakoff’s “Metaphors we live by” (George Lakoff and Johnson 1980a), promoting the essentiality of metaphors in processing and categorizing experiences (Li 2012). In 1987, Lakoff’s “Women, fire, and dangerous things” (Lakoff 1987) and Langacker’s ”Foundation of Cognitive Linguistics” (Langacker 1987) were published and became the ‘bibles’ of cognitive linguistics (Taylor and Wen 2021, p. 1). During that decade, Cognitive Linguistics began to officially transform into a ‘coherent’ and ‘self-conscious’ approach (Dąbrowska and Divjak 2015, p. 2). 

As a flexible framework, Cognitive Linguistics detaches itself from the ‘reductionist’ perception of language (Langacker 2009, p. 167). Instead of viewing language as composed from minimal units, constructions are understood to be the basic units of language (Janda and Dickey 2017). In doing so, it is evident that such flexibility can be translated into Cognitive Linguistics’ focus on continuity rather than on crisp and sharp distinctions (Janda and Dickey 2017). Along with such consistency, it is possible to see two commitments highlighted in most of its literature, in which a common thread needs to run through relevant works to be acknowledged within cognitive linguistics. Lakoff (Lakoff 1990) identifies these as the cognitive commitment and the generalization commitment. According to the first, research adhering to the Cognitive Linguistics enterprise should consider collectively and interdisciplinarily what is available about general cognitive principles and not limit its agendas to linguistic ones only. The second entails concentrating on general principles of language that can be generalized over all aspects of human language. 

With this in mind, it is of key significance to highlight a terminological distinction often made in relevant literature between Cognitive Linguistics (with a capitalized C) and cognitive linguistics (uncapitalized c) (Geeraerts and Cuyckens 2007). The first is only one of the various approaches to carry out a generalized sense of cognitive linguistics, which entails that the second is more of an encompassing umbrella term. From this perspective, research working on natural languages with a mental perception of language such as generative grammar or artificial-intelligence-based linguistic investigation are all part of the cognitive linguistics enterprise. Taylor and Wen (Taylor and Wen 2021) acknowledge the same distinction but classify the capitalized term as a micro approach while the uncapitalized is a macro one. 

Yet, even within the micro approach to Cognitive Linguistics, different orientations can be identified. For instance, Winters and Nathan (Winters and Nathan 2020) contend to the presence of two geographical versions of Cognitive Linguistics, a North American version and a European one. The former bears strong affiliations with Rosch’s categorization and prototype theories (Rosch 1975; Rosch et al. 1976), which heavily impacted Lakoff’s aforementioned pioneering works. The latter, on the other hand, can be linked to what Geeraerts (Geeraerts 1997) classifies as pre-structuralist notions such as polysemy, and onomasiological and semasiological research. These two versions should not be perceived as dichotomies but rather should confirm the flexible continuity-based theorization underlying Cognitive Linguistics discussed earlier. All in all, then, it is possible to conclude this section by highlighting what Croft and Cruse (Croft and Cruse 2004) identify as the three fundamental hypotheses upon which Cognitive Linguistics is based:

- Language is not an autonomous cognitive faculty;
- Grammar is conceptualization; and
- Knowledge of language emerges from language use (Croft and Cruse 2004, p. 1).

### 1.2. The Scope of Cognitive Linguistics

Based on the previous discussion, Wen and Taylor (Taylor and Wen 2021, pp. 2–5) classify the relevant literature on Cognitive Linguistics into seven major categories in which diverse and perhaps competing orientations might overlap. First, there is Cognitive Linguistics research that is phenomenology-based; drawing on Husserl (1859–1938), this orientation calls for examining things as they are and incorporate fundamental veins in Cognitive Linguistics. These include prototype theory and categorization theory (Rosch 1975) as well as conceptual metaphor and metonymy theories (Lakoff and Johnson 1980a), embodied realism and cognitive pragmatics. Some recent examples of this are Wen and Fu’s (Wen and Fu 2021) examination of categorization as an ubiquitous component in reality and Zaifert’s (Zeifert 2022) elaboration of prototype theory. Second, there is the Gestalt-psychology-based orientation which influenced Lakoff’s “Linguistic gestalt” (Lakoff 1977), Langacker’s “Foundations of cognitive grammar” (Langacker 1987) and Fillmore, Kay and O’Conner’s construction grammar (Fillmore et al. 1988) as well as varieties of constructional approaches to cognitive grammar (Langacker 2009). It also encompasses Talmy’s cognitive semantics such as his work on main verb properties (Leonard Talmy 2016) as well as on force dynamics (Leonard Talmy 2018) which does not examine reality but rather its conceptualization (De Mulder 2021). Thus, it is possible to see a common thread running in most of cognitive linguistics research; rather than mirroring reality objectively, language actually imposes its structure on the world (Geeraerts and Cuyckens 2007), promoting intersubjectivity instead (Langacker 2014).

Construction grammar, in particular, has received a special interest in cognitive linguistics literature, which merits further elaboration. It was developed originally by Fillmore, Kay, and O’Conner (Fillmore et al. 1988). The basic premise in such theorization stems from the thesis that grammar can be modelled in constructions rather than through rules and words. A construction in that sense refers to a conventional language pairing of form and meaning; i.e., idiomatic expressions in particular (Evans et al. 2007). Expressions such as kick the bucket or throw in the towel demonstrate such case since their meanings cannot be inferred from the meanings of their individual parts; instead, their meaning is stored as a whole—as a construction—within the linguistic competence of its users. Goldberg (Goldberg 2005) later developed the theory further based on its original thesis (Fillmore et al. 1988) as well as the work of Lakoff (Lakoff 1977; Lakoff and Thompson 1975; Lakoff and Johnson 1980b). Doing so, she extended the theory to incorporate regular constructions, in addition to the idiomatic irregular ones identified in previous thesis while utilizing cognitive concepts like polysemy and metaphor (Evans et al. 2007). Goldberg also modified the notion of construction to be positioned within a lexicon-grammar continuum (Goldberg 2005). 

Cognitive Linguistics is known to be a prominent example of sematic-based linguistic theorization (Winters and Nathan 2020); however, the third orientation reveals a popular trend in recent research inclined towards pragmatics and discourse (Taylor and Wen 2021) in which Lakoff and Turner’s cognitive poetics (Lakoff and Turner 1989), cognitive stylistics, and conceptual blending theory are all examples of this. Representations of discourse are also a salient category in this orientation. This is primarily due to the ‘intricate and inherent’ relation between linguistic units on one hand and discourse on the other; thus highlighting the potential role of context in molding and supporting its interpretation (Langacker 2001b, p. 143). An up-to-date example of this trend is Attardo’s work on the theory of humor from a cognitive linguistics perspective (Attardo 2021) in addition to Hart’s work on the link between cognitive linguistics and critical discourse analysis (Hart 2013). 

Another trend that has been reflected in relatively recent research is due to calls for the social turn (Croft 2009; Harder 2010) in Cognitive Linguistics; that is cognitive sociolinguistics research. The need for such affiliation between cognitive linguistics and sociolinguistics has been intensified (Langacker 2016) due to the increasing usage-based inspiration within Cognitive Linguistics enterprise (Kristiansen and Dirven 2008) and its expanding works on cognitive ideology and cognitive lexical variation research. For instance, Koller’s (Koller 2008) examination of corporate mission statements reveals some cognitive ideological constructions that fall under this category. By the same token, Geeraerts (Geeraerts 2008) explores how a relevant key cognitive construct, prototypes, are linked to Putnam’s (Putnam 1975) socially based notion of stereotypes. A parallel affiliation is also present in the fifth orientation; connecting between Cognitive Linguistics and psycholinguistics. Despite being relatively a novel field (Taylor and Wen 2021), it draws heavily on image schemas (Johnson 1987), in which human sensory-perceptual experience is fundamentally structuring everyday life (Tay 2021). This field offers an expanding arena for examining how figurative language, in all its forms, is inevitable in language processing, usage-based language acquisition, and lexical growth. This can be exemplified by Li’s (Li 2012) work on learning idioms through image schemas and conceptual metaphors and Bergen’s (Bergen 2015) detailed elaboration on embodiment as a central concept encompassing the ubiquitous relation between mind and body. 

The sixth orientation targets cognitive historical linguistics as well as contrastive linguistic research which has flourished recently (Hartmann 2021). By highlighting the diachronic aspect of linguistic theory, it encompasses works on linguistic variation such as Aldokhayel’s work on Classical Arabic case making (Aldokhayel 2021) or Janda and Dickey’s work on Salvic languages (Janda and Dickey 2017). It also encompasses works that are on a wider cross-cultural and cross-linguistic perspective, as in Belkhir’s work on the novice area of proverbs (Belkhir 2021), Midor’s (Midor 2019) work on women’s cross-cultural metaphorical representations of grief and child loss, and Zhou’s (Zhou 2018) examination of cross-linguistic dog connotations. Finally, Taylor and Wen (Taylor and Wen 2021) highlight a seventh orientation as applied cognitive linguistics, which further explores all the practical applications of the Cognitive Linguistics enterprise. In so doing, research within fields of language acquisition and pedagogy as well as translation and ideology is prominently included here. Similarly, research within multimodality (Hart 2016; Ruth-Hirrel and Wilcox 2018), along with works on sign languages (Siyavoshi 2019) also fall under this category. In fact, applications of Cognitive Linguistics have been extended to non-linguistic research too, Hurtienne (2014) for instance, utilizes implications of image-schematic metaphor to the field of user interface design. 

What this classification does not acknowledge, however, is the potential of corpus linguistics tools in Cognitive Linguistics literature, which could be justified on the grounds that corpus linguistics is often presented as an interdisciplinary methodology within linguistic investigation. Since its early days, Cognitive Linguistics has had access to vast corpora (e.g., the Brown Corpus in the 1960s) (Wong 2012). However, some criticize that period for focusing primarily on introspection-based theory-building rather than empirical data (Divjak et al. 2016). Over the course of the next decades, however, this has changed drastically, inspiring Janda (Janda 2013) to dub this fundamental shift in its literature as the quantitative turn.

Corpus linguistics has a substantial potential to research within the cognitive linguistic enterprise. This is primarily due to the fact that corpora can offer linguistic evidence that is based on authentic and natural language use and are inherently deeply rooted in language usage (Arppe et al. 2010). Even prior to the aforementioned quantitative turn that took place a decade ago, such potential has been acknowledged and theorized within aspiring attempts; thus, resulting in an edited volume by (Gries and Stefanowitsch 2006) presenting works by prominent cognitive linguists with corpus-based motivations. The book offered rigid research of where cognitive and corpus linguistics meet and flourish. For instance, Newman and Rice (2009) utilized corpus tools to investigate transitivity schemas associated with the English verbs eat and drink while Lemmens (2009) approached the cognitive-corpus linguistics affiliation from an experimental stance. This affiliation continued to consolidate as more empirical and experimental research within quantitative designs is published; only to name a few (Blumenthal-Dramé 2016; Gries 2014; Luo 2018; Stefanowitsch 2011). 

What is offered by these experimental cognitive-corpus linguistics applications are attempts to support the indirect links between cognition and linguistic data with ‘converging evidence’ (Arppe et al. 2010, p. 6) in a way that allows for the replication of research design and procedure across different experimental conditions. Consistent with the quantitative turn identified earlier, some recent works with cognitive linguistic literature adhere to experimental approaches to analyzing cognitively based linguistic evidence. While such experimental trend can be detected across diverse fields within cognitive linguistics research, works relating to first language acquisition and second language learning have been prominent in experimental research. Tyler, for instance, offered both theoretical backgrounding as well as solid experimental application in a book investigating second language learning with some pedagogical implications (Tyler 2012). By the same token, some other experimental cognitive works such as Aajami (2018) and Taylor and Wen (2021) examined the semantic schemas of English polysemous propositions such as to, for, and at. 

This experimental vein reveals a strong connection to data-driven research within cognitive linguistics. In data-driven approaches, data are used to offer guidelines to the process of policymaking and decision makers to improve the feasibility of having an informed and strategic decision. In light of this, it does not come as a surprise that literature within second language pedagogy research also utilizes such take. Kilimci, for instance, offers pedagogical implications to inform second language learning of prepositions (Kilimci 2017). Others, such as Brdar-Szabó and Brdar, advocate that data-driven approaches within cognitive linguistics could benefit from the affiliation with other introspection-based approaches (Brdar-Szabó and Brdar 2012). Through a contrastive cross linguistic study, they argue for the need for obtaining converging evidence from both theoretical and empirical inspirations. 

In short, Divjak, Levshina, and Klavan classify all these topics within Cognitive Linguistics as revolving around three axes, with each axis exhibiting unbalanced distribution of research in one direction over the other (Divjak et al. 2016). There is the time dimension, exhibiting more synchronic investigations than diachronic ones. There is the linguistic diversity dimension, revealing more research examining one language (mostly English), over that investigating many languages. Finally, there is the modality dimension exhibiting more research centered around written language over multimodal research.

### 1.3. Scientific Contributions for Cognitive Linguistics

As stated in the previous discussion, the second half of the 1980s was quite critical in establishing Cognitive Linguistics as a major field within linguistic enquiry. This was ignited in 1989, when René Dirven organized a symposium in Duisburg, Germany, which was renamed latter as the ‘the First International Cognitive Linguistics Conference’ and resulted in two major landmarks in Cognitive Linguistics history (Nerlich and Clarke 2007). At that conference, the International Cognitive Linguistics Association (ICLA) (Midor 2019) was established and the decision was made to publish a new journal, Cognitive Linguistics (Cognitive Linguistics n.d.), and a monograph series, Cognitive Linguistics Research, both by De Gruyter Mouton in Germany. The first issue of Cognitive Linguistics appeared in 1990, and it continues to lead current research in the field until present time while being indexed in Web of Science WoS (Social Sciences Citation Index SSCI—Arts and Humanities Citation Index AHCI) with an impact factor (IF) of 1.55 in 2020. The ICLA organizes its biennial international conference and is also affiliated with more than 14 organizations all over the world such as the Spanish Cognitive Linguistics Association, the UK Cognitive Linguistics Association and the Discourse and Cognitive Linguistic Society of Korea. Aside from its primary journal and monograph search, the ICLA sponsors many additional publications such as the Annual Review of Cognitive Linguistics (Review of Cognitive Linguistics n.d.) and Cognitive Linguistics in Practice (Cognitive Linguistics in Practice n.d.); both of which are published by John Benjamins in the Netherlands. The first issue of the annual review appeared in 2003 and it also continues until present time; but it was renamed Review of Cognitive Linguistics in 2010. This journal is also indexed in WOS (SSCI—AHCI) with an IF of 0.41 in 2020. Other international journals in WOS include Language and Cognition (SSCI—AHCI, IF: 1.33), Pragmatics and Cognition (SSCI—AHCI, IF: 0.38) CogniTextes (Emerging Source Citation Index ESCI), Journal of Cognitive Science (ESCI) and Constructions and Frames (ESCI). 

### 1.4. Purpose of the Study

The present study attempts to document the rise and scope of cognitive linguistics as it has transformed from its “revolutionary” status to a firmly “established” one (Nerlich and Clarke 2007, p. 592). It no longer possesses a radical image as these transformations are happening year by year (Dabrowska 2016). Instead, it projects itself as an interdisciplinary approach to modern day linguistics, adapting to more empirical and usage-based investigations. That said, this documentation of the rise and development of cognitive linguistics is inclusive of past (i.e., reviewing literature of the development of the field), present (i.e., bibliometric and scientometric review of existing literature), and providing insights into the directions of cognitive linguistics through analysis of past literature and mapping domains of existing literature.

## 2. Materials and Methods

The discipline of scientometrics focuses on the “study of artifacts; one examines not science and scholarship but the products of those activities” (Glänzel and Schoepflin 1994, p. 491). Research in scientometrics examines “the quantitative aspects of the production, dissemination and use of scientific information with the aim of achieving a better understanding of the mechanisms of scientific research as a social activity” (Chellappandi and Vijayakumar 2018, p. 6). This type of research does not necessarily improve published knowledge. It has been shown in several studies that “the task of determining quality papers is especially difficult in BIS [bibliometrics, informetrics and scientometrics] due to the very heterogeneous origin of the researchers” (Egghe 1994, p. 390). Nonetheless, the primary objective of such studies remains to “reveal characteristics of scientometric phenomena and processes in scientific research for more efficient management of science” (Parkinson 2011, p. 1).

The use of scientometric indicators is used to guide such studies. A measure may address one or more elements (e.g., publication, citations and references, potential, etc.) or may indicate a type (e.g., quantitative, impact) (Parkinson 2011). Among the concepts commonly used in such studies is “mapping knowledge domains” which refers to creating “an image that shows the development process and the structural relationship of scientific knowledge”—using maps that are “useful tools for tracking the frontiers of science and technology, facilitating knowledge management, and assisting scientific and technological decision-making” (Huang et al. 2021, p. 6201). Research in this field is increasingly inclusive, including all fields of study, rather than restricting itself to medical, health, and pure science settings (Sooryamoorthy 2020). The present study examines cognitive linguistics as a branch of interdisciplinary linguistics that integrates with other fields like linguistics and cognitive sciences.

### 2.1. Measures

Both bibliometrics and scientometrics conceive of studies as tools for assessing knowledge production in a field (e.g., cognitive linguistics). Knowledge databases (e.g., Scopus, WOS, and Lens) are the most common source of bibliometric data (Birkle et al. 2020; Burnham 2006; Pranckutė 2021; Penfold 2020). The majority of scientometric indicators are generated by scientometric software. In our current study, we used CiteSpace 5.8.R3 (Chen 2014) and VOSviewer 1.6.18 (van Eck and Waltman 2022). Table 1 shows that both bibliometric and scientometric criteria were used in this study.

### 2.2. Data Collection and Sample

For data retrieval, we used three databases: Scopus, WOS, and Lens. These databases were included for a number of reasons. WOS and Scopus index publications according to their criteria only (Pranckutė 2021; Birkle et al. 2020; Burnham 2006). Moreover, Lens provides a broader range of data unavailable in Scopus or WOS (Penfold 2020).

Searches were conducted on Thursday, 16 June 2022. Language limitations were not taken into consideration as long as titles, abstracts, and keywords were provided in English. Since there were very few results available in our list of other languages, we manually verified the results to ensure that they were relevant. We considered articles, reviews, book chapters, books, dissertations, conference proceedings (full papers), and early access publications of these types for this study. Table 2 lists the strings for the three databases and other specifications.

We assessed the growth and size of the cognitive linguistics field by using the concept of “cognitive linguistics.” Since the term cognitive linguistics results in a large number of results, we did not use specific search terms to narrow the results or find specific topics related to cognitive linguistics. The above search strings are suggested to be useful for searching for information about cognitive linguistics after a preliminary Google search and review of our prior knowledge in the field.

### 2.3. Data Analysis

Data analysis began with several steps taken. We exported Scopus data in three different formats: Excel sheets, RIS (i.e., Research Information Systems) files for CiteSpace, and CSV files for VOSviewer. In order to meet CiteSpace’s requirements, the RIS file was converted to WOS. Additionally, WOS data were retrieved in two formats: text documents converted to Excel sheets for bibliometric analysis, and plain text documents for CiteSpace and VOSviewer. For the bibliometric analysis and for VOSviewer, Lens data were retrieved in two formats: CSV and a full record CSV.

We removed duplicate documents using CiteSpace and Mendeley before using CiteSpace for analysis. In order to conduct the bibliometric analysis, Excel was used. Using Excel, we generated tables for the citation reports and converted them to figures.

We used both programs’ default settings for scientometric analysis. A different visualization was done for each database, including overlays, network visualizations, and density visualizations. Scoups and WOS analyses were conducted in three stages: cooccurrence analysis by author keywords, co-citation analysis by source, and co-citation analysis by cited author. There were four analyses conducted for Lens: cooccurrence by author keyword, citation by author, citation by source, and citation by document. The following analyses were performed in CiteSpace for Scopus and WOS: co-citations by document (references), co-citations by cited authors, and occurrence (keywords). Summaries of the results are presented as narratives, cluster summaries, maps, and burst tables.

## 3. Results

There are two sections in the results. A bibliometric analysis of cognitive linguistics is presented in the first section. These indicators were derived based on data retrieved from the Scopus, WOS, and Lens databases. Bibliometric indicators include publications by year, top 10 countries, universities, journals, publishers, subject areas, and authors. A description of scientometric indicators pertaining to cognitive linguistics development is presented in the second section. To analyze these indicators, CiteSpace and VOSviewer were used. Among the indicators are co-citations, citations, and cooccurrences.

### 3.1. Bibliometric Indicators for the Study of Cognitive Linguistics

#### 3.1.1. Overview of Cognitive Linguistics Studies from Scopus, Web of Science, and Lens

We retrieved 2380 documents from Scopus, 1732 from WOS, and 9911 from Lens, all related to cognitive linguistics. Each database was based on data from 1983 to 2022, 1987 to 2022, and 1969 to 2022. This time span was selected based on the availability of the data. In other words, the starting data were automatically determined based on identifying documents in cognitive linguistics since 1969 and the ending data was based on the available papers on the three databases by the day of making the search. A total of 1485 articles, 239 review articles, 319 book chapters, 163 books, and 174 conference papers were included in Scopus. Among the documents from the WOS were 1326 articles, 34 review articles, 100 book chapters, 100 books, 13 early access papers, and 357 proceedings papers. From Lens, 7, 243 articles, 2133 unclassified, 716 book chapters, 464 books, 185 dissertations, 21 preprints, and 149 conference proceedings were included. These documents were mainly written in English, but included documents in Russian, Spanish, Portuguese, German, Italian, French, Chinese, and other languages. As the analysis is based on title, keywords, abstract, and references, all of these include this information in English. Inclusion of this data was considered to avoid bias towards English-published data.

**Figure 1:**
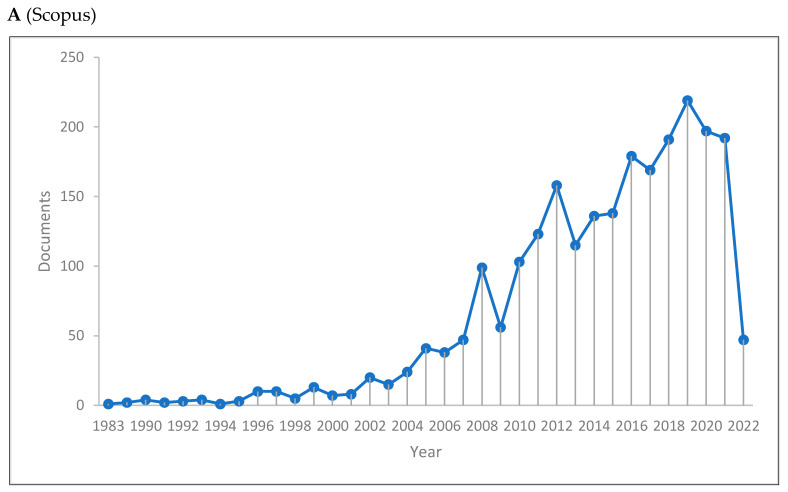

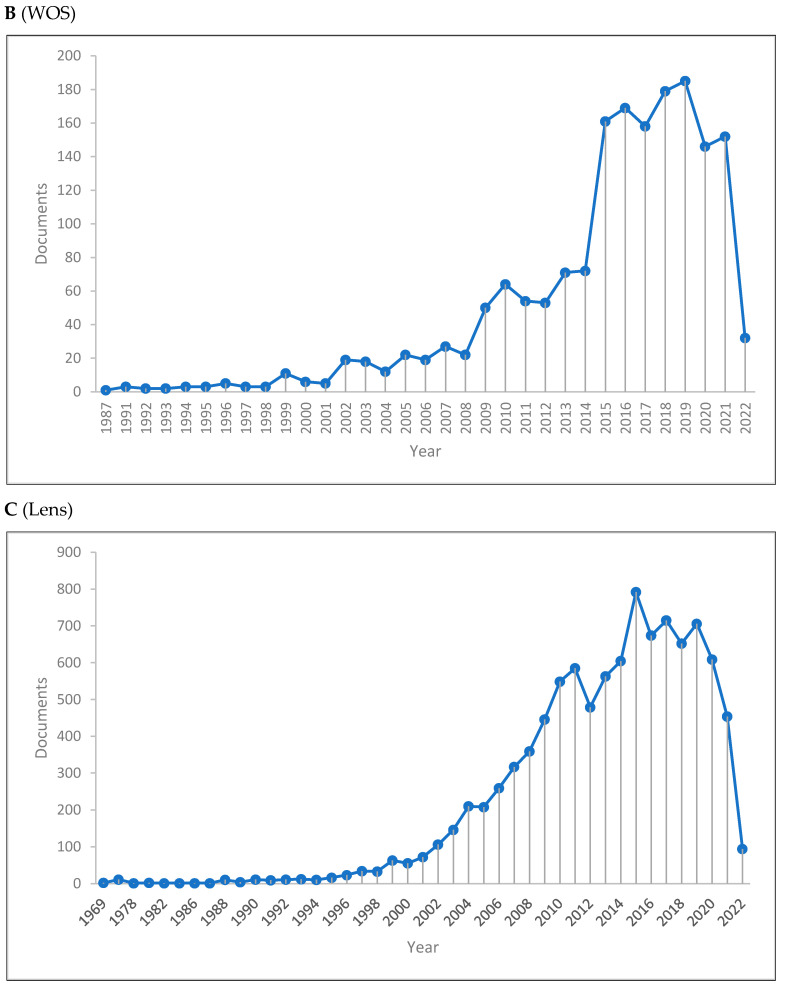
Knowledge production size of cognitive linguistics by year.

Figure 1A–C shows the length of production by year for the three databases. There has been a significant rise in knowledge production in cognitive linguistics, reaching its peak in 2019 in Scopus with 219 publications, 2019 in the WOS with 185 publications, and 2015 in Lens with 792 publications. The Scopus database has a range of 1–219 publications, the WOS has a range of 1–185 publications, and Lens has a range of 1–792 publications. Further, of 14,023 included documents, only 350 documents were published before 2000. This supports the increase of knowledge production in cognitive linguistics, although the data for the current year (i.e., 2022) were only until 16 June. The lowest number of publications occurs the previous years. The last two decades have seen a rise in the production of cognitive linguistic knowledge.

#### 3.1.2. Production of Cognitive Linguistics Research by Country and University

**Figure 2:**
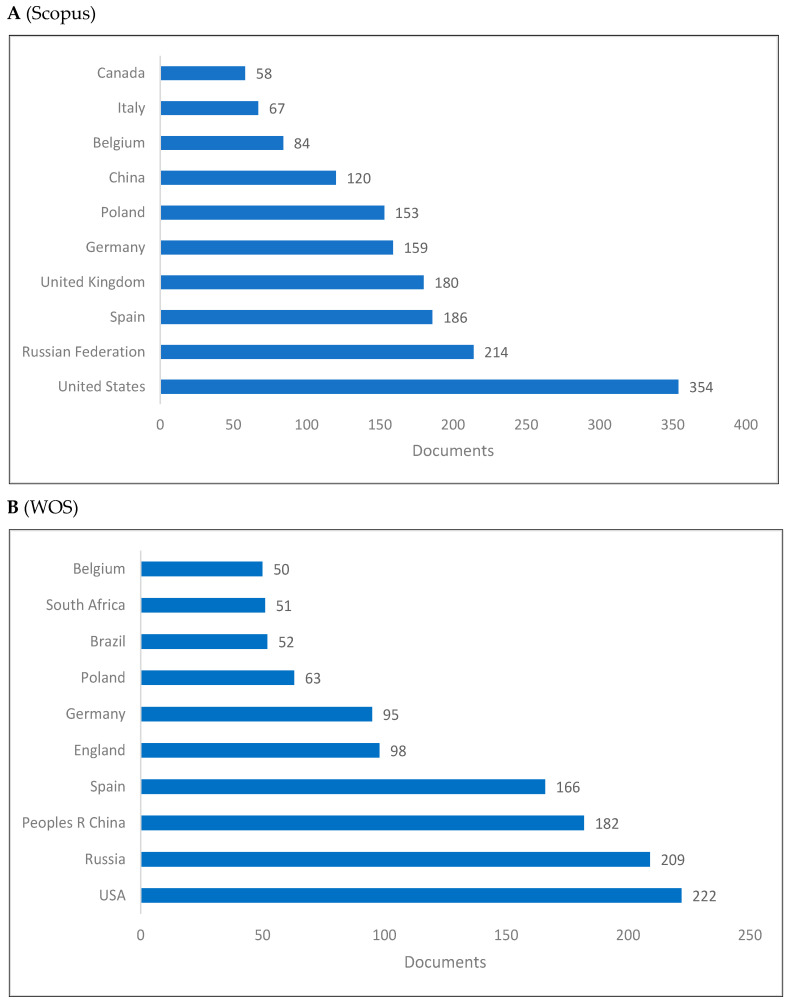

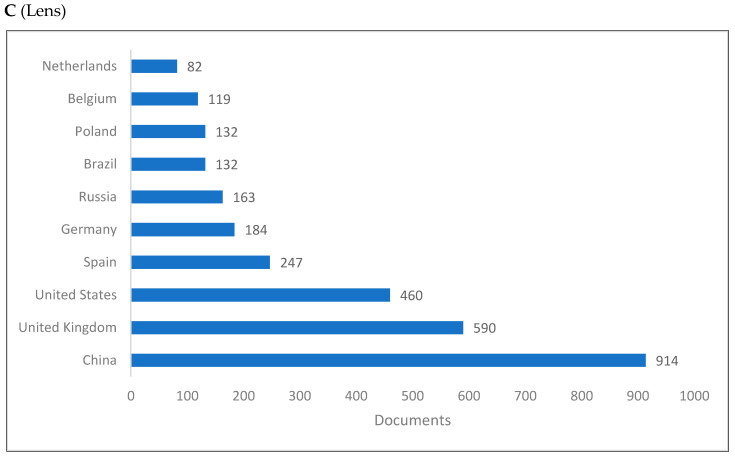
Knowledge production size of cognitive linguistics by country.

Figure 2A–C shows the top 10 producing countries for knowledge related to cognitive linguistics. There is a variable distribution of rankings for the top 10 countries producing knowledge in cognitive linguistics across the three databases. Although the US and Russia rank first and second in Scopus and WOS, China ranks first and the UK ranks second in Lens. Except for China from Asia, Brazil from South America, and South Africa from Africa, most of these top 10 countries are geographically located in Europe or North America.

**Figure 3:**
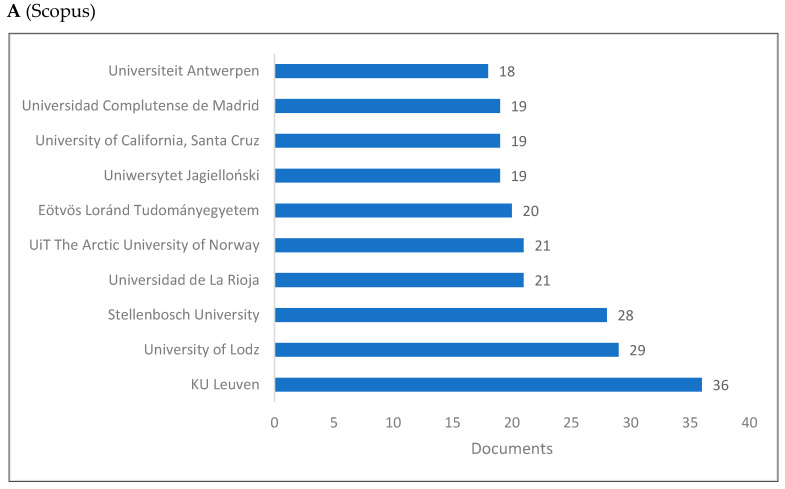

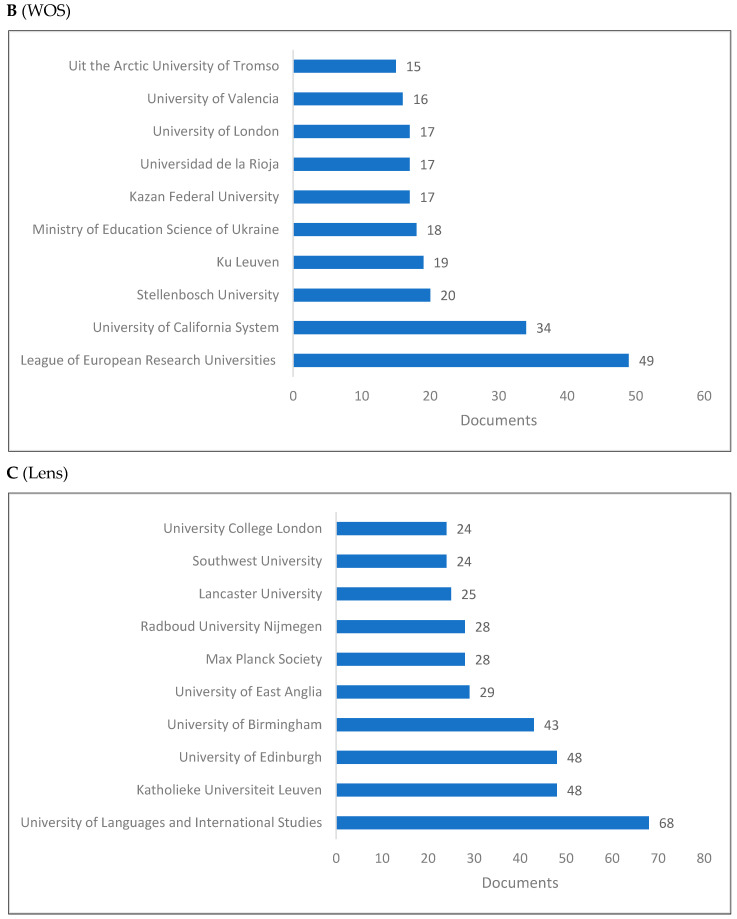
Knowledge production size of cognitive linguistics by university/research center.

Figure 3A–C presents the top 10 universities and/or research centers producing knowledge in cognitive linguistics. The majority of these universities are based in European countries, with a few universities located in Russia and the United States as well.

#### 3.1.3. Production of Cognitive Linguistics Research by Journal and Publisher

**Figure 4:**
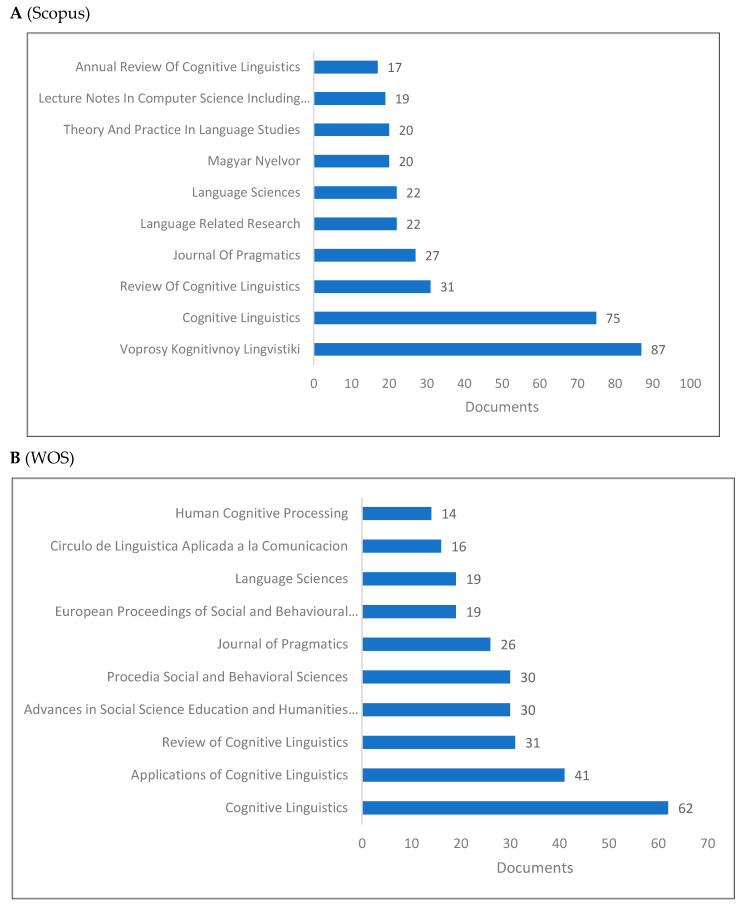

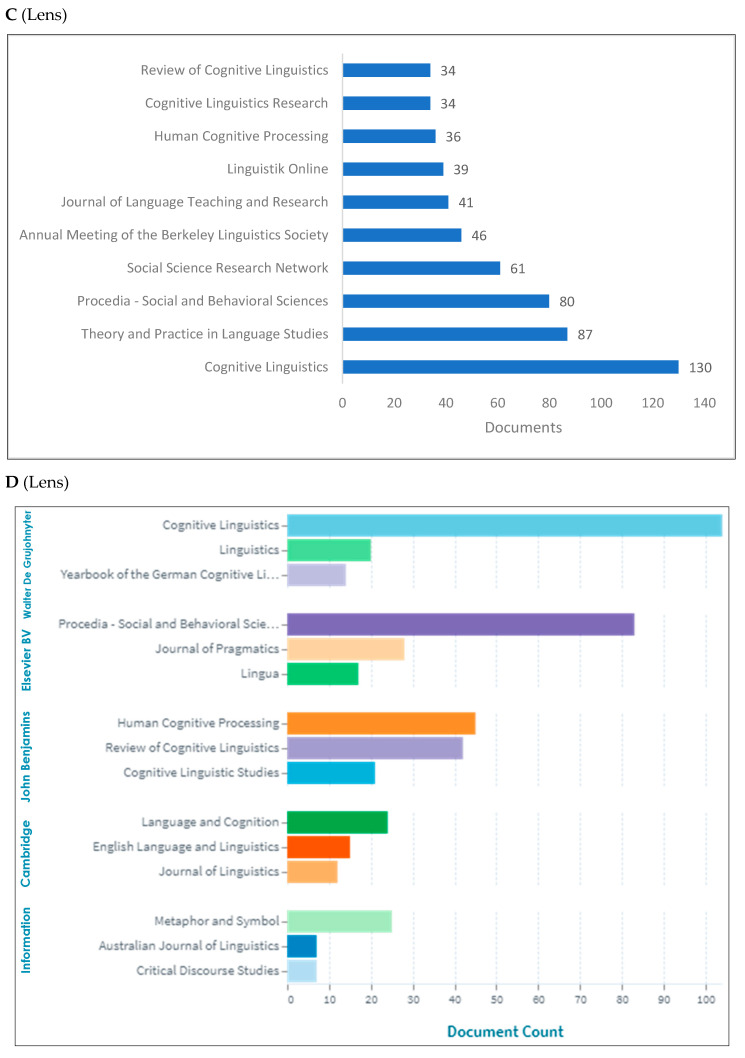
Knowledge production size of cognitive linguistics by journal.

Figure 4A–D demonstrates the top 10 journals publishing research in cognitive linguistics. Several of the top journals are closely related or even include “cognitive” in their titles while the rest are related to cognitive sciences, computer science, language studies, and pragmatics. An extended list of journals based on publishers is shown in Figure 4D. In this list, we can see several journals related to cognitive linguistics and cognitive sciences, as well as social sciences and linguistics journals.

**Figure 5:**
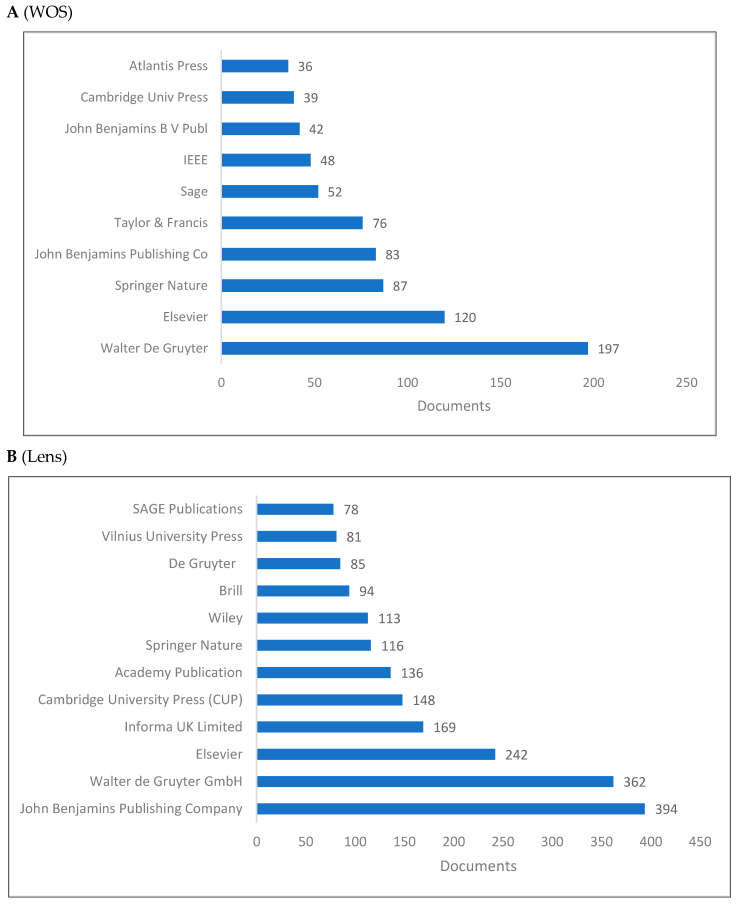
Knowledge production size of cognitive linguistics by publisher.

Figure 5A,B shows the list of top 10 publishers for knowledge in cognitive linguistics. Due to Scopus’ lack of publisher information, these lists are limited to the WOS and Lens databases. In both databases, Walter De Grujohnyter, Elsevier, and Springer Nature appear to be the top three publishers. Despite the difference in rankings, the same publishers are available in both databases.

#### 3.1.4. Production of Cognitive Linguistics by Research Area, Keywords, and Cooccurrence

**Figure 6:**
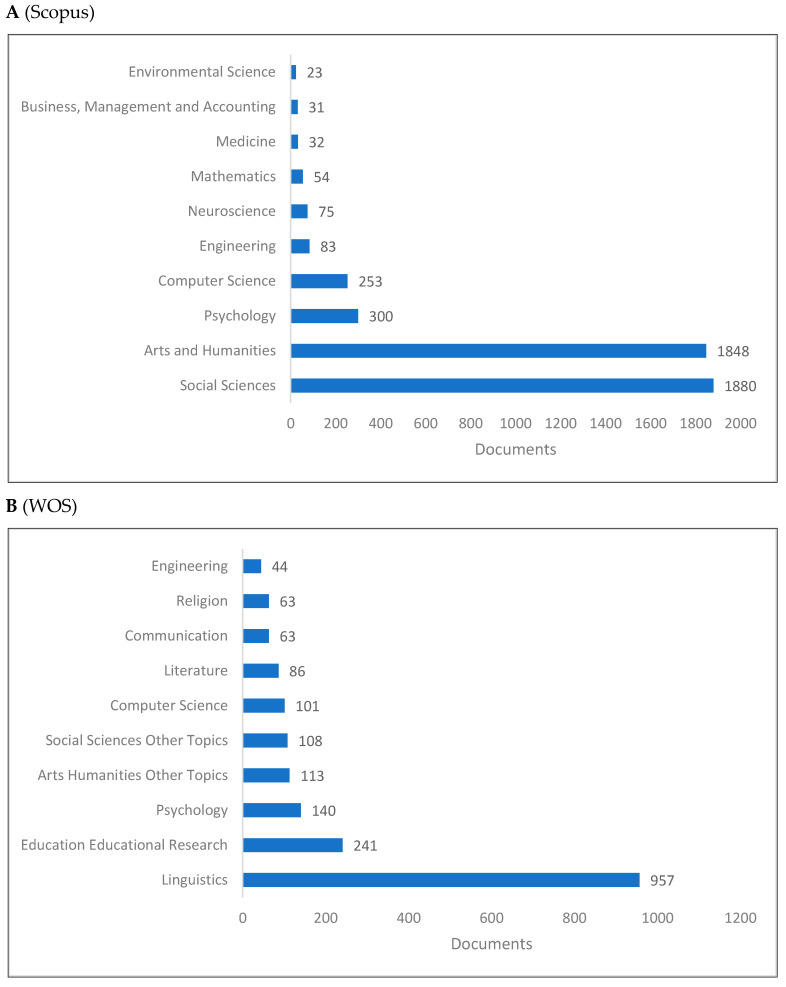

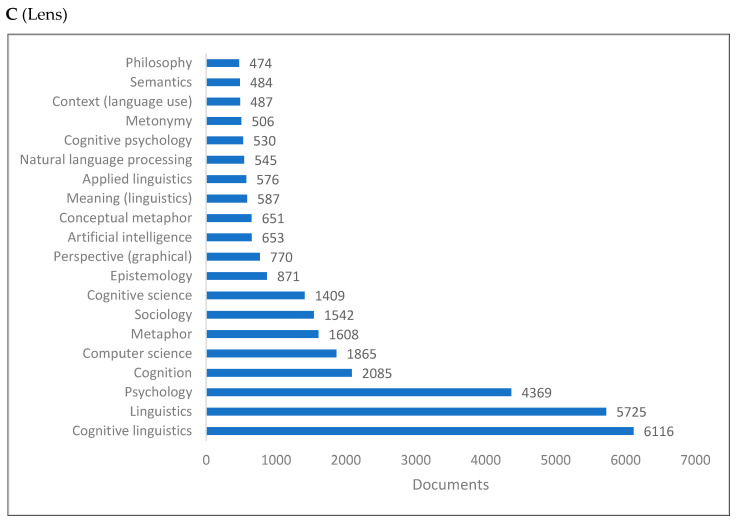
Knowledge production size of cognitive linguistics by research area.

Cognitive linguistics is part of interdisciplinary linguistics, which studies language and cognitive science, but also integrates these fields with many other fields (Figure 6A–C). According to Figure 6A, social sciences, arts and humanities, psychology, and computer science account for the majority of publications in cognitive linguistics. Research in cognitive linguistics focuses primarily on linguistics, educational research, psychology, and arts and humanities, as shown in Figure 6B. Further confirmation can be found in Figure 6C, where cognitive linguistics, linguistics, psychology, and cognition are presented as the top four fields of study published in cognitive linguistics. More specific cognitive linguistics fields are presented on Lens (e.g., metaphors, natural language processing, metonymies, semantics).

#### 3.1.5. Production of Cognitive Linguistics by Authors

**Figure 7:**
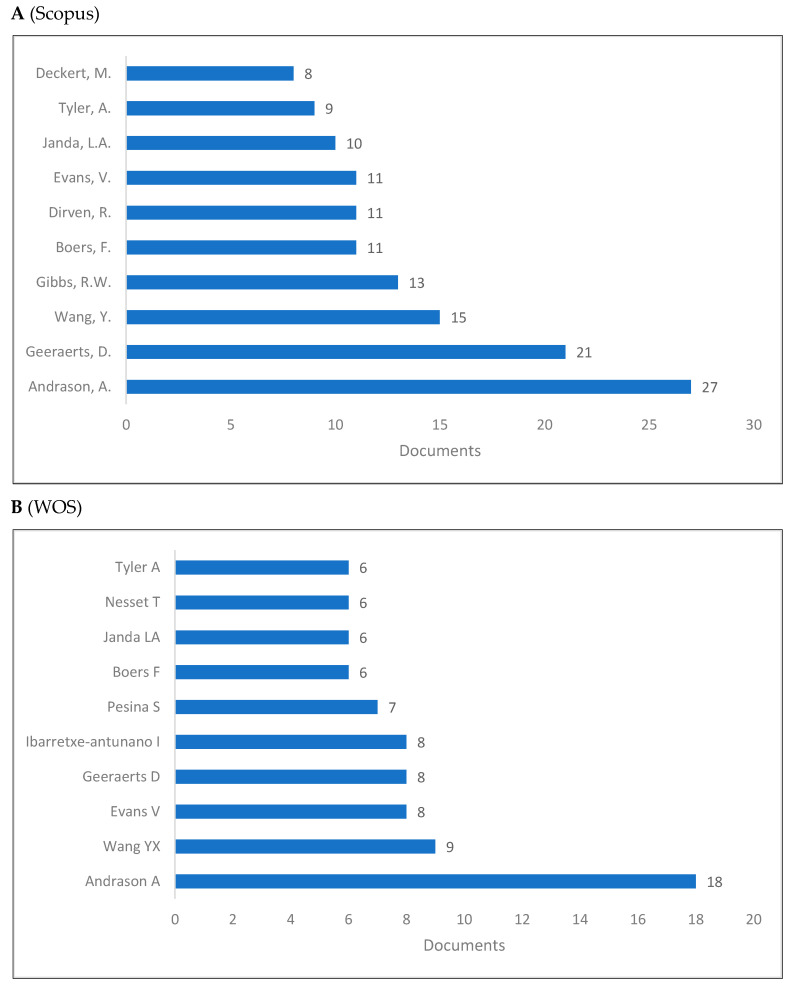

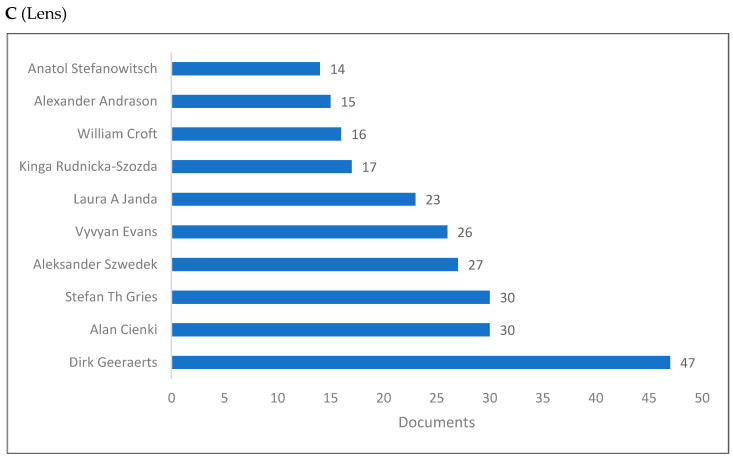
Knowledge production size of cognitive linguistics by author.

In cognitive linguistics, contributions are not necessarily limited to a certain number of authors and a single article may constitute a contribution. Nevertheless, we aimed to show the authors who contributed more knowledge to cognitive linguistics (Figure 7A–C). It can be seen that Andrason (Andrason 2016), Wang (Wang and Berwick 2012), and Geeraerts (Geeraerts 2008) are among the top positions, but this ranking is altered when the Lens database is considered.

### 3.2. Scientometric Indicators for the Study of Cognitive Linguistics

#### 3.2.1. Overview of Cognitive Linguistics Studies from Scopus, Web of Science, and Lens

We present here a scientometric analysis of the data that were retrieved from Scopus, WOS, and Lens databases. Several concepts, authors, references, and emerging trends are highlighted.

**Figure 8:**
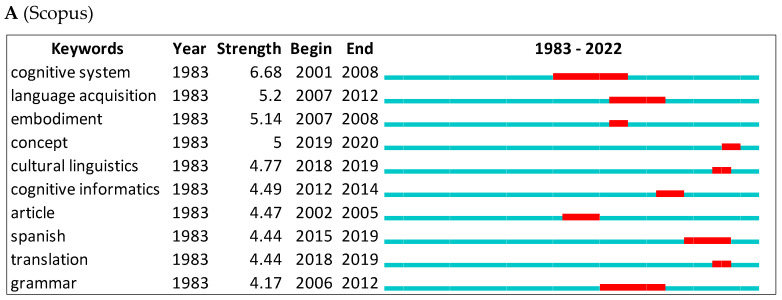

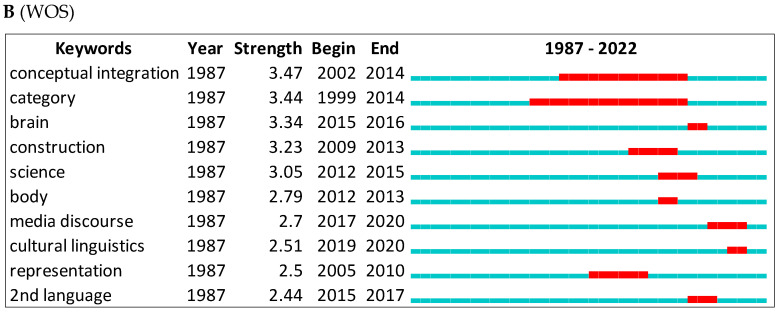
Top 10 keywords with the strongest citation bursts.

Using CiteSpace, we first show the top keywords with the strongest citation bursts from Scopus and WOS (Figure 8A,B). All research is represented by the green line. Red lines indicate the beginning and end of bursts. The word with the strongest citation burst in Scopus is cognitive system (=6.68) between 2001 and 2008, and conceptual integration (=3.47) between 2002 and 2014 for the WOS. Citation bursts vary by database. In the WOS, we can see second language and body, but in Scopus, we can see concept and grammar.

**Figure 9:**
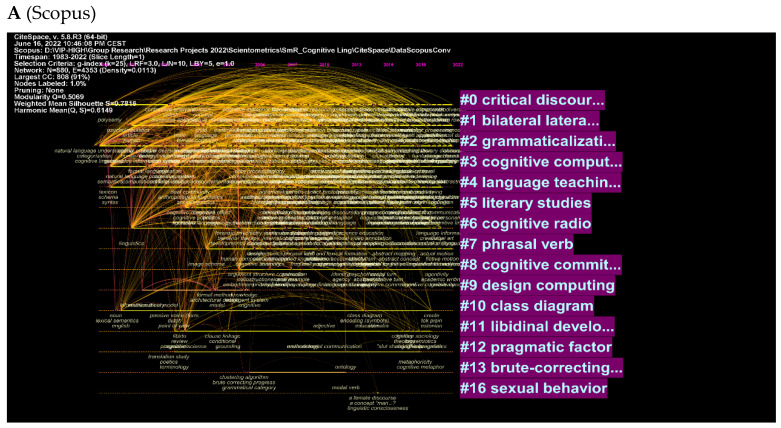

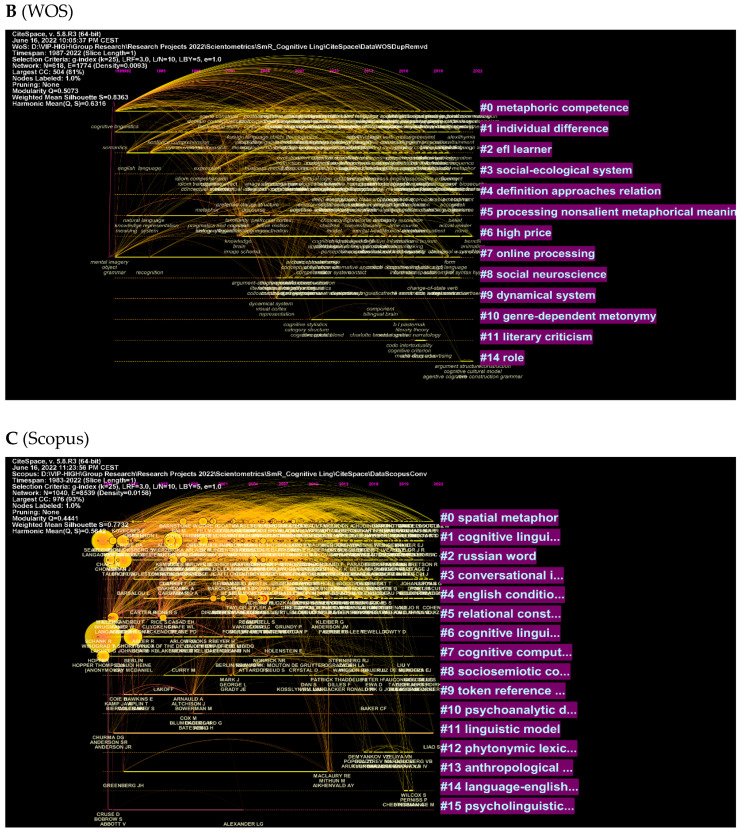

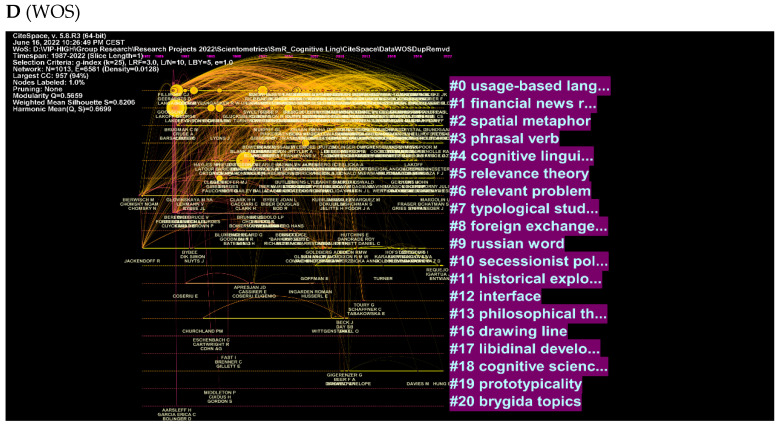
Top keywords, cited authors, and clusters.

Visualizations of clusters and authors further illustrate these concepts (Figure 9A–D). Among the most explored topics in cognitive linguistics, Figure 9A shows critical discourse, language teaching, and grammaticalization. Figure 9B shows more specific concepts such as metaphoric competence, individual differences, and English teaching and learning. A list of the most cited authors is shown in Figure 9C,D, along with the topics that were searched when these authors were cited. Among these topics are spatial metaphors, cognitive linguistics, and Russian word. (See Figure 9C). WOS includes other words such as usage-based language, relevance theory, etc. (See Figure 9D). More importantly, these figures are better understood in terms of the list of the clusters that were identified automatically based on the quantity of research sharing the same cluster. Next to each cluster is the intensity indicating how much research is produced in relation to this particular cluster. Some clusters are skipped automatically by the software to show the most the relevant clusters that are very specific to the use of the concept ‘cognitive linguistics’.

**Figure 10:**
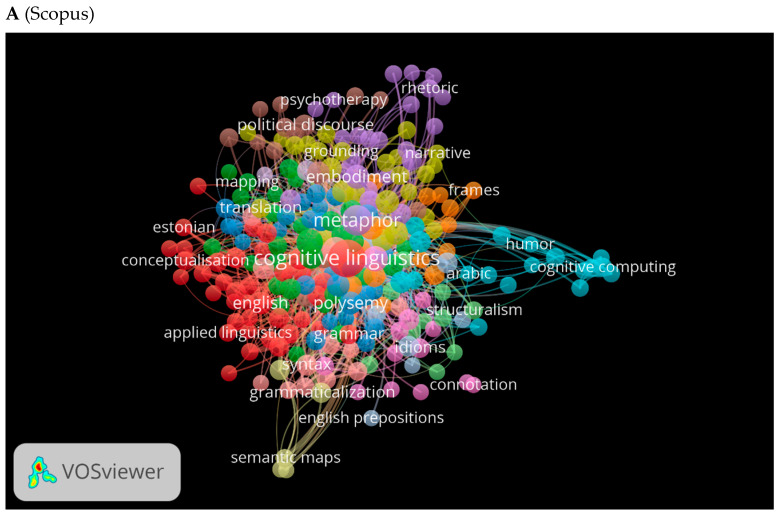

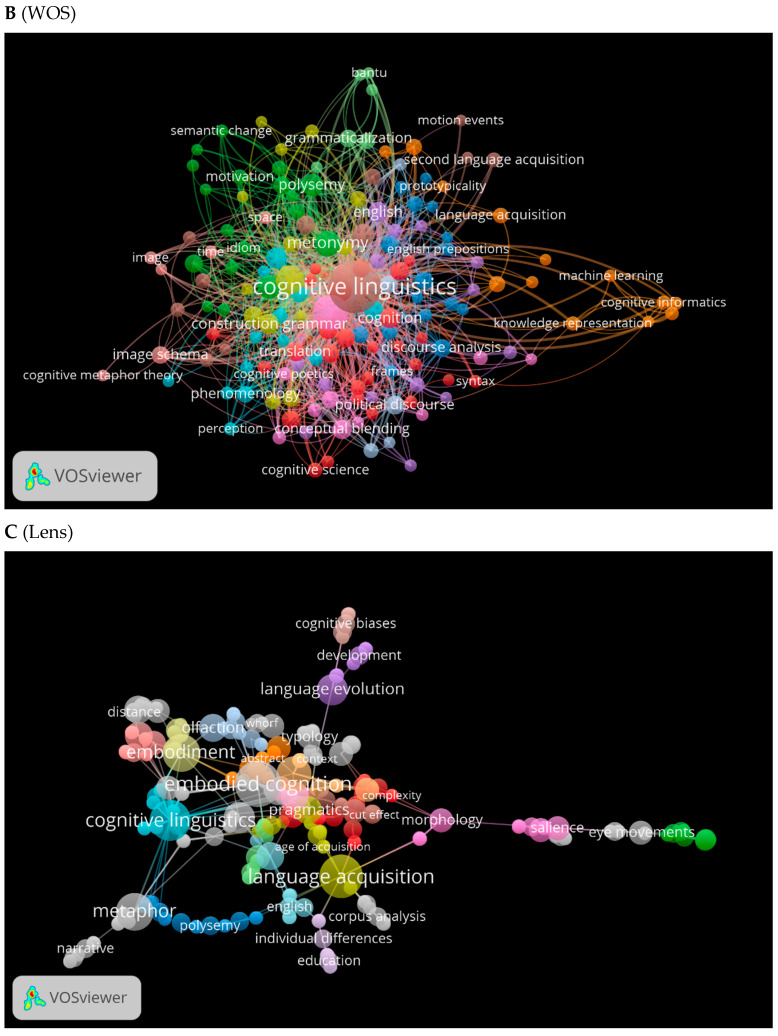
Cooccurrence by author keywords network visualization.

Another important factor is the co-occurrence of used keywords. The three databases were visually mapped using VOSviewer to show the occurrence of the most commonly used cognitive linguistics keywords (Figure 10A–C). In cognitive linguistics, each color represents a particular direction. The larger the size of the font or the circles, the more research is produced in that area of research. Red indicates cognitive linguistics, blue shows cognitive computing, and purple depicts rhetoric (See Figure 10A). Depending on the database, these colors may change. As shown in Figure 10B, green indicates topics related to metonymy, pink to cognitive linguistics, and orange to machine learning. Figure 10C displays metaphor and narrative keywords in gray.

**Figure 11:**
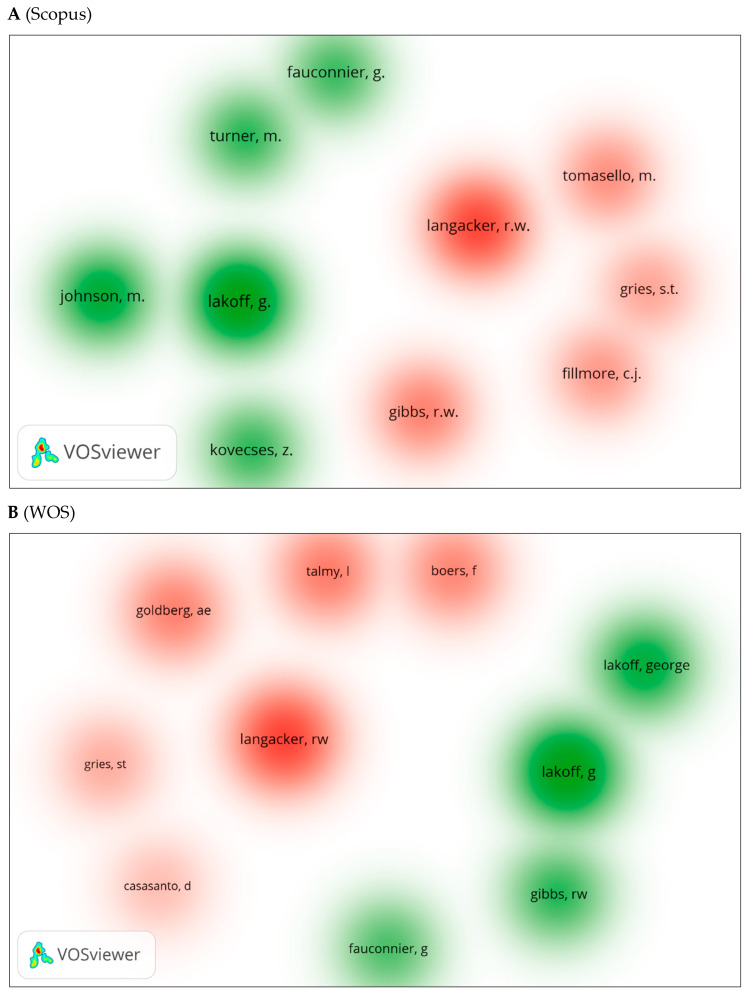

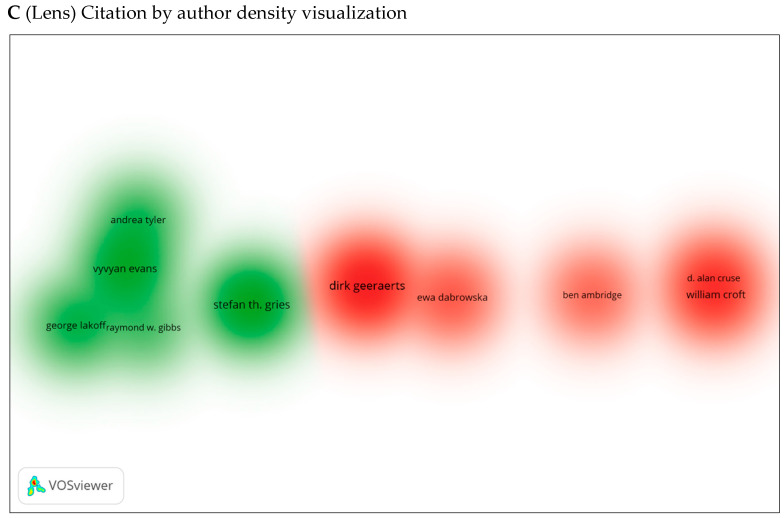
Co-citation by cited author density visualization.

We generated three visual network maps using VOSviewer for co-citation and citation by author (Figure 11A–C). Each color represents a co-citation or citation network between authors. A larger circle indicates that the author has been co-cited or cited more frequently. The same authors appear in all three databases, whether for co-citations or citations. These include Langacker (Langacker 2012), Lakoff (Lakoff 1990), Gibbs (Gibbs 1999), etc.

**Figure 12:**
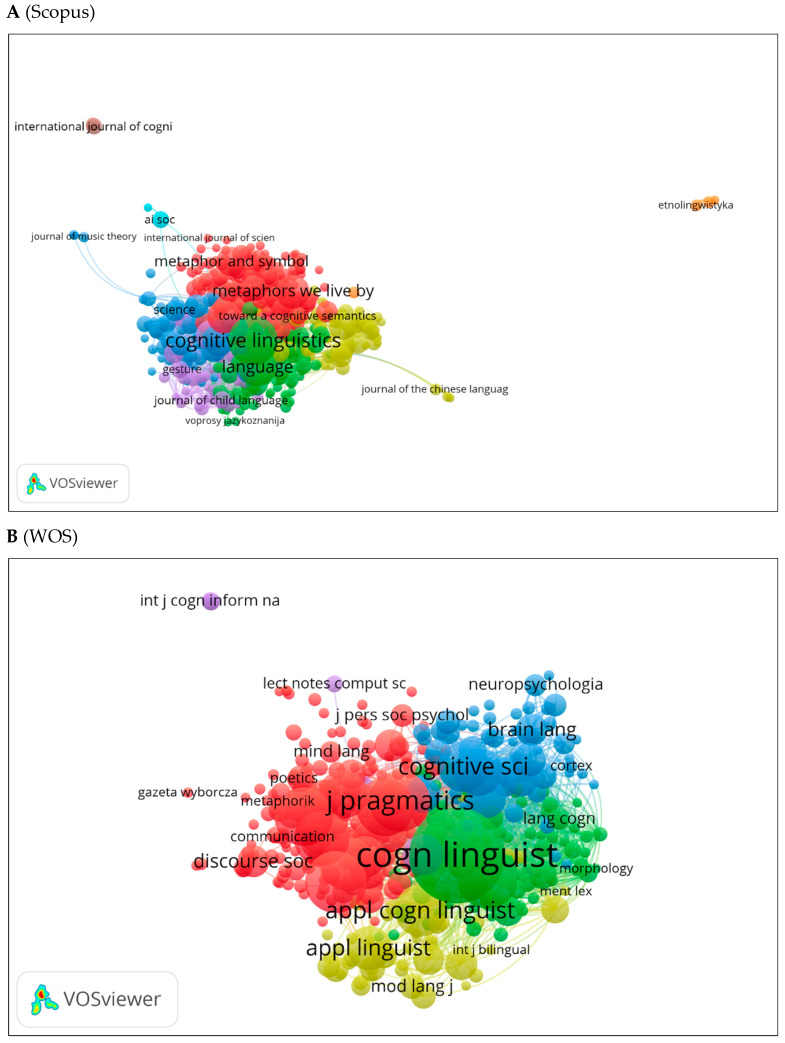

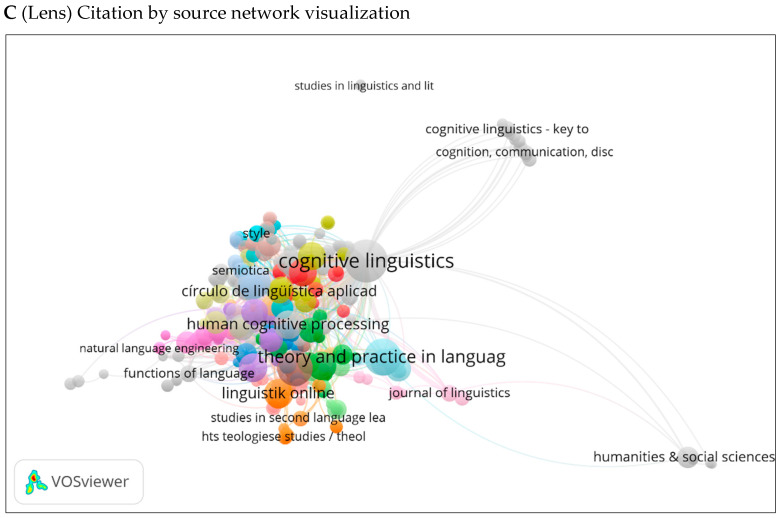
Co-citation by source network visualization.

VOSviewer was used to generate three visual network maps of co-citations and citations by source (Figure 12A–C). Colors represent networks of co-citations or citations. An increased circle size indicates a greater number of co-citations. In Figure 12A, Cognitive Linguistics appears to be the most co-cited source. Figure 12B shows similar results using the WOS database with other notable journals (e.g., Cognitive Science, Journal of Pragmatics). The citation network for journals is shown in Figure 12C. These include Human Cognitive Processing, Theory and Practice in Language, etc.

Using the bibliometric data provided in Scopus, WOS, and Lens, we exported the citations reports and reported the top 10 cited works. We then merged them, removed duplicates, and presented citation counts for each (Table 3). The most cited document are “Cognitive Linguistics” in Scopus with 1614 citations, “The brain’s concepts: The role of the sensory-motor system in conceptual knowledge” in the WOS with 1306 citations, and “Philosophy in the flesh: the embodied mind and its challenge to Western thought” in Lens with 5703 citations.

#### 3.2.2. Impact of Research on Cognitive Linguistics by Clusters, Citation Counts, Citation Bursts, Centrality, and Sigma

##### Clusters

The network is divided into 16 co-citation clusters (See Table 4 for details). The largest 6 clusters are summarized as follows. The largest cluster (#0) has 168 members and a silhouette value of 0.708. It is labeled as spatial metaphor by LLR, cognitive linguistics by LSI, and business communication (1.07) by MI. The most relevant citer to the cluster is “The Cambridge handbook of cognitive linguistics” (Dancygier 2017).

The network is divided into 19 co-citation clusters. (See Table 4 for details.). The largest 7 clusters are summarized as follows. The largest cluster (#0) has 188 members and a silhouette value of 0.69. It is labeled as usage-based language by LLR, cognitive linguistics by LSI, and special volume (1.73) by MI. The most relevant citer to the cluster is “Cognitive linguistics and its place in history of linguistics” (Ibarretxe-Antuñano 2013).

##### Citation Counts

In Scopus, the top ranked item by citation counts is Lakoff (Lakoff 1989) in Cluster #6, with citation counts of 1206. The second one is Langacker (Langacker 1989) in Cluster #1, with citation counts of 581. In the WOS, the top ranked item by citation counts is Lakoff (Lakoff 1991) in Cluster #1, with citation counts of 646. The second one is [Anonymous] (1999) in Cluster #4, with citation counts of 570. The remaining top 10 citation counts in cognitive linguistics can be found in Table 5.

##### Bursts

**Figure 13:**
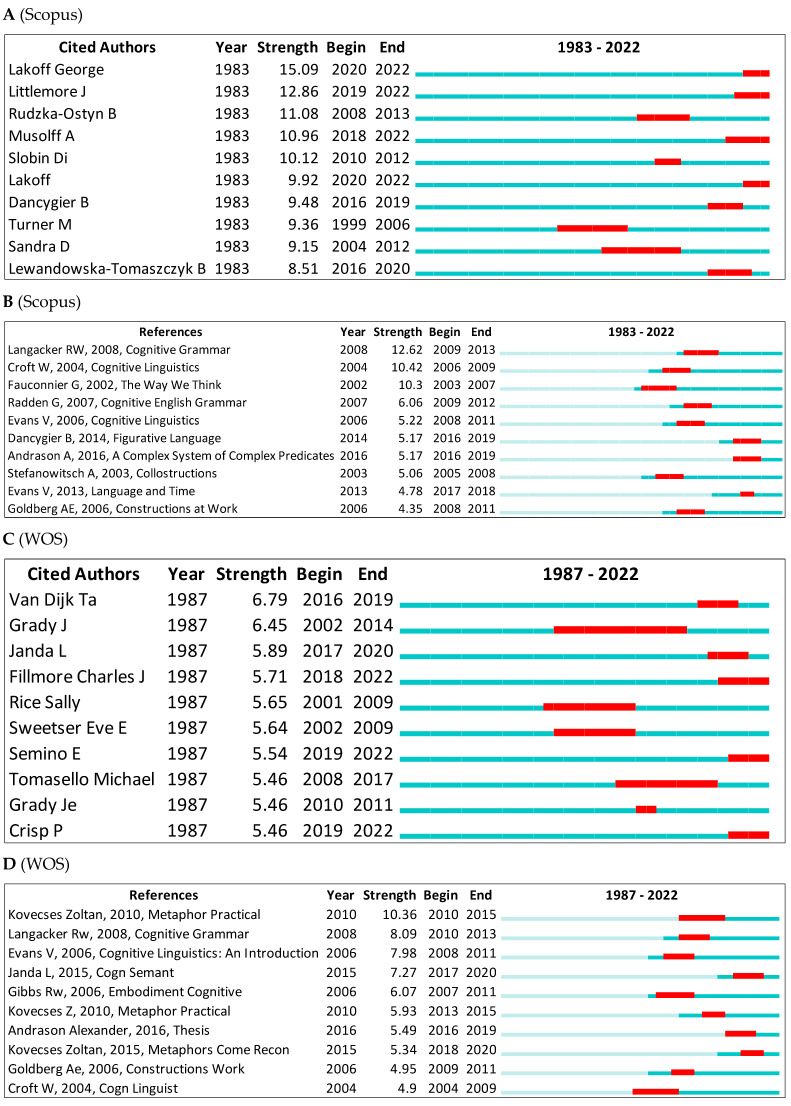
Top 10 cited authors and references with the strongest citation bursts.

In Scopus, the top ranked item by bursts is Lakoff (Lakoff and Johnson 2020) in Cluster #8, with bursts of 15.09. The second one is Littlemore (Low et al. 2007) in Cluster #4, with bursts of 12.86. In the WOS, the top ranked item by bursts is Van Dijk (Van Dijk 2011) in Cluster #1, with bursts of 6.79. The second one is Grady (Grady 2005) in Cluster #2, with bursts of 6.45. See Table 6 and Figure 13A–D for the remaining top 10 detected bursts in cognitive linguistics. The green colour represents the whole period of citation and red colour indicates the detected citation period.

##### Centrality

In Scopus, the top ranked item by centrality is Chomsky (Chomsky 1990) in Cluster #2, with centrality of 136. The second one is Jackendoff (Jackendoff 1995a) in Cluster #5, with centrality of 111. In the WOS, the top ranked item by centrality is Boers (Boers 2001) in Cluster #3, with centrality of 104. The second one is Gibbs (Gibbs 1996) in Cluster #1, with centrality of 103. For a list of the remaining 10 central top authors in cognitive linguistics, please refer to Table 7.

##### Sigma

In Scopus, the top ranked item by sigma is Chomsky (Chomsky 1990) in Cluster #2, with sigma of 0.00. The second one is Jackendoff (Jackendoff 1995a) in Cluster #5, with sigma of 0.00. In the WOS, the top ranked item by sigma is Boers (Boers 2001) in Cluster #3, with sigma of 0.00. The second one is Gibbs (Gibbs 1996) in Cluster #1, with sigma of 0.00. See Table 8 for the remaining top 10 works with sigma value in cognitive linguistics.

## 4. Discussion

This study examined cognitive linguistics as a branch of interdisciplinary linguistics that integrates with other fields such as linguistics and cognitive sciences. This purpose has been achieved by reviewing the rise of cognitive linguistics and by presenting both bibliometrics and scientometrics indicators of cognitive linguistics. Two sections comprise the results of the study. The first section presents the bibliometric indicators including publications by year, top 10 countries, universities, journals, publishers, subject areas, and authors. The second section presents the scientometric indicators, including citation, co-citation, and cooccurrence indicators. With reference to bibliometric indicators, seven key points are discussed: (1) knowledge production in cognitive linguistics increased in the last two decades reaching its peak in 2019 in both Scopus and WOS, and 2015 in Lens; (2) for the top 10 countries producing knowledge, the US and Russia rank first and second in both Scopus and WOS, while China ranks first and the UK ranks second in the Lens; (3) universities in the European countries dominate knowledge production in cognitive linguistics with a few universities in Russia and the US; (4) in both WOS and Lens, Cognitive Linguistics journal ranks the first, while it is proceeded by Voprosy Kognitivnoy Lingvistiki in Scopus; (5) Walter De Gruyter appears as the top publisher followed by Elsevier in WOS database, while John Benjamins Publishing Company appears first followed by Walter De Gruyter in Lens; (6) Linguistics, Cognitive Linguistics, Social Sciences, Arts and Humanities are the top subject areas publishing in cognitive linguistics; and (7) Andrason (Andrason 2016), Wang (Wang and Berwick 2012), and Geeraerts (Geeraerts 1991) are the top in the field.

With reference to scientometric indicators, our analysis identified the most cited keywords in Scopus and WOS. In Scopus, the most cited keywords include language acquisition (Achard and Niemeier 2004), embodiment (Johnson and Lakoff 2002), concept and conceptual system (Evans et al. 2007), and cultural linguistics (Sharifian 2017). In WOS, they include conceptual integration (Turner 2010), construction (Turner 2020), category (Evans et al. 2007), brain (Evans 2012), and science (Sinha 2010).

In terms of the largest co-citation clusters, we analyzed data from both Scopus and WOS. In Scopus, the results include cognitive linguistics research, which theoretically and empirically studies language, conceptual systems, and human cognition (Fauconnier 2006), and usage-based linguistics (Behrens 2009). On the other hand, the results in WOS include financial news reporting, i.e., the interplay of language use and journalism, media and society (Catenaccio et al. 2011), and spatial metaphor, for instance, in relation to memory (Roediger 1980).

With regards to the contributions of the most cited authors, Littlemore (Littlemore 2009) comprehensively deals with teaching cognitive linguistics. Both Lakoff and Johnson (Lakoff and Johnson 1980b) and Grady (Grady 1999) discuss conceptual metaphor. Janda (Janda 2015) provides an inclusive overview of cognitive linguistics, while Van Dijk (Van Dijk 2019) outlines the theory of macrostructures.

Using bibliometric data, we pointed out the top 10 cited articles in both Scopus and WOS. They include topics such as the Invariance Hypothesis (Lakoff 1990), metaphor and metaphorical thoughts (Lakoff 1992), subjectification (Langacker 1990), the mental spaces framework (Fauconnier and Sweetser 1996), cognitive grammar (Langacker 2001a), and motion events (Leonard Talmy 1991).

Lastly, we performed a sigma analysis to determine the top-ranked items in both Scopus and WOS. These include the notion of surface filter which explain certain properties of infinitival constructions (Chomsky and Lasnik 1977), the theoretical foundations of Construction Grammar (Goldberg 2005), the application of the concept of Invariance Hypothesis (Brugman 1990), levels of mental representation (languages of the mind) (Jackendoff 1995b), and learners’ retention of idioms through imagery processing experiment (Boers 2001).

## 5. Conclusions

### 5.1. Practical Implications

Whenever it comes to scientometric studies, researchers should be careful in interpreting the results (van Eck and Waltman 2014), no matter how popular this method has become (van Eck et al. 2010; Moral-Muñoz et al. 2020). Ideally, data should be retrieved from multiple sources and avoided being confined to a single database unless well justified (e.g., we used Scopus, WOS, and Lens in this study). For the next step, different tools should be used for the analysis to incorporate various scientometric indicators (e.g., in this study both CiteSpace and VOSviewer were used). Another practical implication is related to the design and conduct of scientometric studies. In this study, we presented a sample study for conducting scientometric studies. We proposed a whole structure for the structure, content, and format to help researchers conduct similar studies in the future using similar structure, content, and format.

### 5.2. Theoretical Implications

This study has at least two theoretical implications. Firstly, linguistic analyses should not be restricted to words, phrases, and concepts, but should include sentence structures, text structures, context structures, and discourse structures, as well as the comparison of data from different languages to assess convergence and/or divergence. As a second point, behavioral evidence should include both linguistic and non-linguistic aspects of communication in order to obtain evidence regarding cognitive processes involving human communication (e.g., (Al-Mansour et al. 2015; Almukhaizeem and Alduais 2015a; Alduais and Almukhaizeem 2015; Almukhaizeem and Alduais 2015b; Alduais 2017)). Furthermore, brain imaging evidence should incorporate multiple linguistic and non-linguistic tasks at all levels (i.e., as mentioned in the linguistic analysis) to provide more concrete evidence of cognitive processes occurring in the brain. In rehabilitation for those with language breakdown caused by brain injury, trauma, or other causes, this last one will play a significant role.

### 5.3. Limitations

Certain limitations of this study could be addressed in future research. For example, the study focused primarily on the presentation and visualization of bibliometric and scientometric indicators without a significant amount of discussion of the scope of cognitive linguistics. Therefore, a scoping review of cognitive linguistics that considers defining and conceptualizing the field may be an appropriate first step for future research. This study also has the limitation of presenting evidence concerning the development of cognitive linguistics. While both bibliometric and scientometric indicators can be used for such objectives, they remain insufficient to assess the quality of the knowledge produced. Consequently, future research should examine the credibility and practicality of the methods used to study cognitive linguistics. One more limitation is that we did not use any additional search keywords that also include cognitive linguistics (e.g., memory, perception, recall, etc.).

## Figures and Tables

**Table 1:** Scientometrics and bibliometric indicators to measure development of Cognitive Linguistics, adapted from (Alduais et al. 2022).

Element	Definition/Specification/Retrieved Data	Database/Software					
Indicator		Scopus		WOS		Lens	
Bibliometric							
Year	Production size by year	√		√		√	
Country	Top countries publishing in the field	√		√		√	
University	Top universities, research centers, etc.	√		√		√	
Source	Top journals, book series, etc.	√		√		√	
Publisher	Top publishers	Χ		√		√	
Subject area	Top fields associated with the field	√		√		√	
Author	Top authors publishing in the field	√		√		√	
Citation	Top cited documents	√		√		√	
Scientometric		CiteSpace			VOSviewer		
Betweenness centrality	A path between nodes and is achieved when located between two nodes (Freeman 1979)	√			Χ		
Burst detection	Determines the frequency of a certain event in certain period (e.g., the frequent citation of a certain reference during a period of time) (Kleinberg 2002)	√			Χ		
Co-citation	When two references are cited by a third reference (Chen 2016). CiteSpace provides document co-citation network for references, and author co-citation network for authors. In VOSviewer, co-citation defined as “the relatedness of items is determined based on the number of times they are cited together” (van Eck and Waltman 2022, p. 5). Units of analysis include cited authors, references, or sources.	√			√		
Silhouette	Used in cluster analysis to measure consistency of each cluster with its related nodes (Chen 2014)	√			Χ		
Sigma	To measure strength of a node in terms of betweenness centrality citation burst (Chen 2014)	√			Χ		
Clusters	“We can probably eyeball the visualized network and identify some prominent groupings” (Chen 2014, p. 23).	√			√		
Citation	“The relatedness of items is determined based on the number of times they cite each other” (van Eck and Waltman 2022, p. 5). Units of analysis include documents, sources, authors, organizations, or countries.	√			√		
Keywords	CiteSpace provides co-occurring author keywords and keywords plus. In VOSviewer, co-occurrence analysis is defined as “the relatedness of items is determined based on the number of documents in which they occur together” (van Eck and Waltman 2022, p. 5). Units of analysis include author keywords, all keywords, or keywords plus.	√			√		

**Table 2:** Search strings for retrieving data to measure development of Cognitive Linguistics.

Scopus TITLE-ABS-KEY (“cognitive linguistics”) AND (LIMIT-TO (DOCTYPE, “ar”) OR LIMIT-TO (DOCTYPE, “ch”) OR LIMIT-TO (DOCTYPE, “re”) OR LIMIT-TO (DOCTYPE, “cp”) OR LIMIT-TO (DOCTYPE, “bk”)) Thursday, 16 June 2022, 2380 document results, 1983–2022
WOS “cognitive linguistics” (Topic) and Articles or Proceedings Papers or Book Chapters or Review Articles or Early Access or Books (Document Types) Thursday, 16 June 2022, 1732 document results, 1987–2022
Lens (Title: (“cognitive linguistics”) OR (Abstract: (“cognitive linguistics”) OR Full Text: (“cognitive linguistics”))) Filters: Stemming = Disabled Publication Type = (journal article, unknown, book chapter, book, dissertation, conference proceedings article, conference proceedings, preprint) Thursday, 16 June 2022, Scholarly Works (9911), 1969–2022

**Table 3:** Top cited documents of Cognitive Linguistics using citation reports from Scopus, WOS, and Lens.

No.	Source Title	Citation	Citations by Database		
			Scopus	WOS	Lens
1	A Metaphor-Enriched Social Cognition	(Landau et al. 2010)	455	438	Χ
2	An agent-based conception of models and scientific representation	(Giere 2010)	Χ	123	Χ
3	An integrated theory of language production and comprehension	(Pickering and Garrod 2013)	Χ	Χ	910
4	An introduction to cognitive linguistics	(Ungerer and Schmid 2013)	Χ	Χ	686
5	Cognitive linguistics	(Croft and Cruse 2004)	1614	Χ	1282
6	Cognitive linguistics: An introduction	(Evans and Green 2018)	852	Χ	706
7	Embodiment as a unifying perspective for psychology	(Schubert and Semin 2009)	295	262	Χ
8	Framing, Agenda Setting, and Priming: The Evolution of Three Media Effects Models	(Scheufele and Tewksbury 2007)	Χ	Χ	1705
9	Grammar is grammar and usage is usage	(Newmeyer 2003)	Χ	169	Χ
10	Grounded Cognition: Past, Present, and Future	(Barsalou 2010)	405	367	Χ
11	Literal vs. figurative language: Different or equal?	(Giora 2002)	Χ	134	Χ
12	Multimodal metaphor	(Forceville and Urios-Aparisi 2009)	248	Χ	Χ
13	Philosophy in the flesh: the embodied mind and its challenge to Western thought	(Lakoff and Johnson 1999)	Χ	Χ	5703
14	Prosody in the comprehension of spoken language: a literature review.	(Cutler et al. 1997)	Χ	Χ	814
15	Reading acquisition, developmental dyslexia, and skilled reading across languages: a psycholinguistic grain size theory.	(Ziegler and Goswami 2005)	Χ	Χ	2113
16	Relational Leadership Theory: Exploring the social processes of leadership and organizing	(Uhl-Bien 2006)	Χ	Χ	1171
17	Sociocultural theory and L2: State of the art	(Lantolf 2006)	Χ	205	Χ
18	The brain’s concepts: The role of the sensory-motor system in conceptual knowledge	(Gallese and Lakoff 2005)	1484	1306	1812
19	The Invariance Hypothesis: Is abstract reason based on image-schemas?	(Lakoff 1990)	470	Χ	Χ
20	The role of the right hemisphere in processing nonsalient metaphorical meanings: Application of Principal Components Analysis to fMRI data	(Mashal et al. 2005)	Χ	157	Χ
21	The semantics of English prepositions: Spatial scenes, embodied meaning and cognition	(Tyler and Evans 2003)	340	Χ	Χ
22	Turning the tables: Language and spatial reasoning	(Li and Gleitman 2002)	325	295	Χ

**Table 4:** Summary of the largest clusters in Cognitive Linguistics.

Cluster ID	Size	Silhouette	Label (LSI)	Label (LLR)	Label (MI)	Average Year
Scopus						
0	168	0.708	cognitive linguistics	spatial metaphor (593.54, 1.0 × 10^−4^)	business communication (1.07)	2010
1	158	0.672	cognitive linguistics	cognitive linguistic research (604.68, 1.0 × 10^−4^)	business communication (1.41)	2005
2	154	0.673	cognitive linguistics	Russian word (730.85, 1.0 × 10^−4^)	business communication (1.05)	2006
3	107	0.77	cognitive linguistics	conversational informatics (569, 1.0 × 10^−4^)	business communication (0.48)	2009
4	107	0.745	cognitive linguistics	English conditional (698.61, 1.0 × 10^−4^)	business communication (1.23)	2010
5	63	0.859	cognitive linguistics	relational construction (339.89, 1.0 × 10^−4^)	business communication (0.58)	2000
WOS						
0	188	0.69	cognitive linguistics	usage-based language (564.62, 1.0 × 10^−4^)	special volume (1.73)	2010
1	140	0.7	cognitive linguistics	financial news reporting (540.24, 1.0 × 10^−4^)	special volume (2.7)	2010
2	101	0.716	cognitive linguistics	spatial metaphor (372.1, 1.0 × 10^−4^)	special volume (0.63)	2010
3	98	0.894	cognitive linguistics	phrasal verb (555.48, 1.0 × 10^−4^)	special volume (0.81)	2010
4	75	0.814	cognitive linguistics	cognitive linguistic research (205.26, 1.0 × 10^−4^)	special volume (1.95)	2003
5	68	0.876	cognitive linguistics	relevance theory (316.09, 1.0 × 10^−4^)	special volume (0.52)	2005
6	62	0.966	cognitive linguistics	relevant problem (255.83, 1.0 × 10^−4^)	special volume (0.06)	1999

**Table 5:** Citation counts for Top 10 works in Cognitive Linguistics.

WoS			Scopus		
Citation	Reference	Cluster ID	Citation	Reference	Cluster ID
646	Lakoff (Lakoff 1991)	1	1206	Lakoff (Lakoff 1989)	6
570	[Anonymous], 1999	4	581	Langacker (Langacker 1989)	1
474	Lakoff (Lakoff 1991)	1	424	Fauconnier (Fauconnier and Sweetser 1996)	1
323	Langacker (Langacker 1991)	0	386	Talmy (Talmy 1990)	2
246	Langacker (Langacker 2001b)	0	385	Johnson (Johnson 1992)	6
220	Gibbs (Gibbs 1996)	1	385	Croft (Croft 1991)	2
208	Johnson (Johnson 2014)	1	290	[Anonymous], 1989	7
191	Talmy (Leonard Talmy 1983)	0	286	Geeraerts (Geeraerts 1991)	1
191	Evans (Evans and Green 2006)	3	282	Evans (Evans 2005)	4
190	Kovecses (Kövecses 2004)	1	268	Gibbs (Gibbs 1999)	1

**Table 6:** Detected bursts for top 10 works in Cognitive Linguistics.

WoS			Scopus		
Burst	Reference	Cluster ID	Burst	Reference	Cluster ID
6.79	Van Dijk (Van Dijk 2011)	1	15.09	Lakoff (Lakoff and Johnson 2020)	8
6.45	Grady (Grady 2005)	2	12.86	Littlemore (Low et al. 2007)	4
5.89	Janda (Janda and Dickey 2017)	0	11.08	Rudzka-Ostyn (Rudzka-Ostyn 2003)	4
5.71	Fillmore (Fillmore 1966)	0	10.96	Musolff (Musolff 2011)	0
5.65	Rice (Newman and Rice 2001)	0	10.12	Slobin (Slobin 2003)	4
5.64	Sweetser (Sweetser 2007)	1	9.92	Lakoff (Lakoff 1995)	8
5.54	Semino (Demmen et al. 2015)	1	9.48	Dancygier (Dancygier 2017)	0
5.46	Tomasello (Rakoczy et al. 2008)	0	9.36	Turner (Turner 1999)	0
5.46	Crisp (Crisp et al. 2002)	1	9.15	Sandra (Geudens et al. 2004)	5
5.46	Grady (Grady 2005)	1	8.51	Lewandowska-Tomaszczyk (Lewandowska-Tomaszczyk 2016)	1

**Table 7:** Betweenness centrality for top 10 works in Cognitive Linguistics.

WoS			Scopus		
Centrality	Reference	Cluster ID	Centrality	Reference	Cluster ID
104	Boers (Boers 2001)	3	136	Chomsky (Noam Chomsky 1990)	2
103	Gibbs (Gibbs 1996)	1	111	Jackendoff (Jackendoff 1995a)	5
94	Goldberg (Goldberg and Bencini 2005)	0	102	Brugman (Brugman 1990)	5
92	Langacker (Langacker 1991)	0	100	Tomasello (Tomasello 2003)	4
92	Talmy (Talmy 1995)	0	93	Barlow (Bowker and Barlow 2008)	2
87	Langacker (Langacker 2001b)	0	90	Gibbs (Gibbs 1999)	1
87	Croft (Croft 2005b)	0	86	Boers (Boers 2001)	4
85	Croft (Croft 2005a)	0	86	Barsalou (Barsalou 2000)	1
81	Geeraerts (Geeraerts 1991)	0	84	Johnson (Johnson 1992)	6
80	Chomsky (Chomsky 1987)	6	83	Ortony (Ortony et al. 1990)	1

**Table 8:** Sigma values for top 10 works in Cognitive Linguistics.

WoS			Scopus		
Sigma	Reference	Cluster ID	Sigma	Reference	Cluster ID
0	Boers (Boers 2001)	3	0	Chomsky (Chomsky 1990)	2
0	Gibbs (Gibbs 1996)	1	0	Jackendoff (Jackendoff 1995a)	5
0	Goldberg (Goldberg and Bencini 2005)	0	0	Brugman (Brugman 1990)	5
0	Langacker (Langacker 1991)	0	0	Tomasello (Tomasello 2003)	4
0	Talmy (Talmy 1995)	0	0	Barlow (Bowker and Barlow 2008)	2
0	Langacker (Langacker 2001b)	0	0	Gibbs (Gibbs 1999)	1
0	Croft (Croft 2005b)	0	0	Boers (Boers 2001)	4
0	Croft (Croft 2005a)	0	0	Barsalou (Barsalou 2000)	1
0	Geeraerts (Geeraerts 1991)	0	0	Johnson (Johnson 1992)	6
0	Chomsky (Chomsky 1987)	6	0	Ortony (Ortony et al. 1990)	1

## Data Availability

The data presented in this study are available on request from the first author.
